# Editorial

**Published:** 1839-06-08

**Authors:** 


### THE MEDICAL EXAMINER.

PHILADELPHIA, JUNE, 8 1839.

---

We learn that Drs. Wood and Bache are preparing for the press a new edition of the United States Dispensatory. This will be the fourth edition, and, like the preceding, will probably be a large one. This excellent work is now generally introduced throughout the United States, and is equally necessary to physicians and druggists.

---

We give in the present number a translation of Professor Gerdy's Discourse at the Royal Academy of Medicine, on the distinction between the nerves of sensation and motion. This forms a part of a series of lectures and debates upon the subject, which have recently taken place at the Academy. Dr. Gerdy is well-known as a physiologist. We publish his lecture because it is in opposition to the commonly received notions upon this subject, and, although the grounds of his reasoning are not incontrovertible, it is well to hear both sides of the question.

The translation is literally correct, and made with great care; but we must apologize to our readers for a number of Gallicisms which will be found throughout it. The translator is a young physician, well acquainted with the French language, and with the subject; but, from want of practice in translating, he has rendered some passages so literally, that their meaning is somewhat obscured.

